# Supplementary material for: Ecological Factors Associated with the Distribution of Bemisia tabaci Cryptic Species and Their Facultative Endosymbionts
Source: Insects. 2023 Mar 2;14(3):252. doi: 10.3390/insects14030252 (PMC10053707; doi:10.3390/insects14030252)
Supplement: Supplementary file 1 [file insects-14-00252-s001.zip › insects-2236499-supplemenmtary figures.pdf]

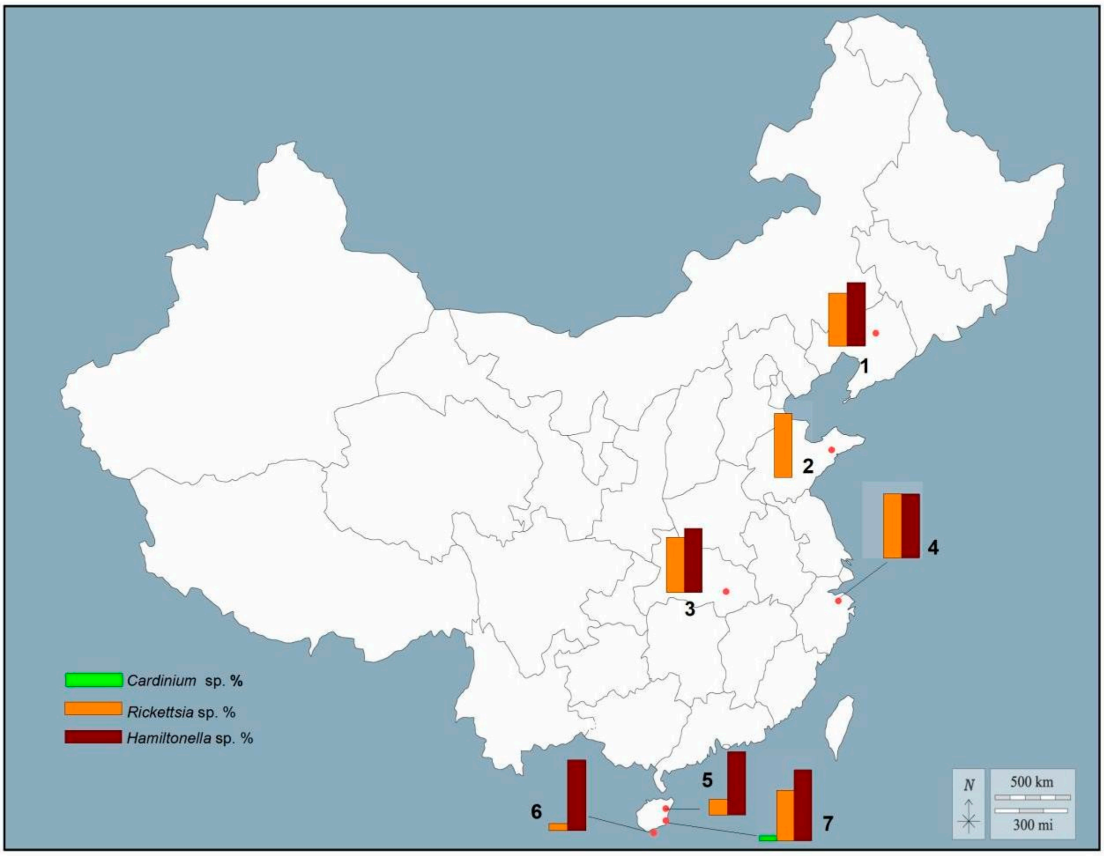

Figure S1. Infection frequency of three facultative endosymbionts in *Bemisia tabaci* MEAM1 populations across China. Green, yellow and red bars represent the infection frequencies of *Cardinium* sp., *Rickettsia* sp., and *Hamiltonella* sp., respectively. Numbers on the map correspond to locality numbers in Table S4 in the supplemental material.

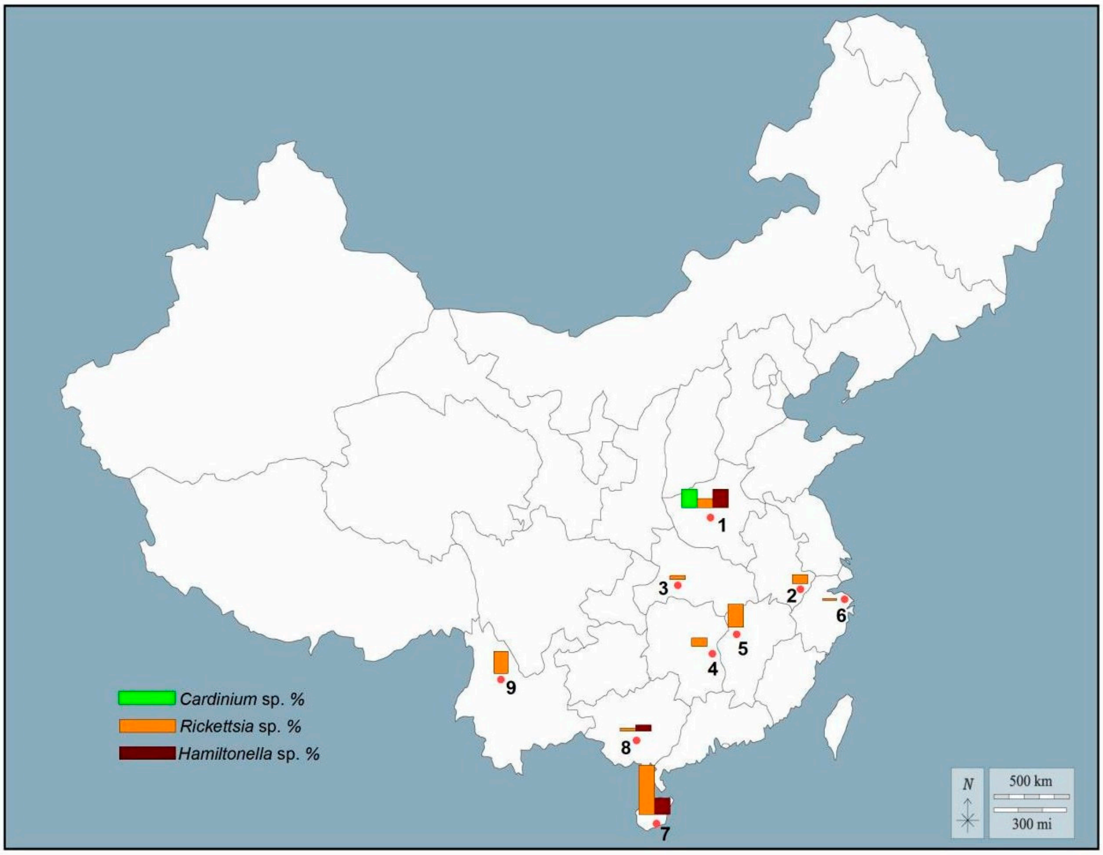

Figure S2. Infection frequency of three facultative endosymbionts in *Bemisia tabaci* native cryptic species across China. Green, yellow and red bars represent the infection frequencies of *Cardinium* sp., *Rickettsia* sp.and *Hamiltonella* sp., respectively. Numbers on the map correspond to locality numbers in Table S5 in the supplemental material.
