# Supplementary material for: Ecological Factors Associated with the Distribution of Bemisia tabaci Cryptic Species and Their Facultative Endosymbionts
Source: Insects. 2023 Mar 2;14(3):252. doi: 10.3390/insects14030252 (PMC10053707; doi:10.3390/insects14030252)
Supplement: Supplementary file 1 [file insects-14-00252-s001.zip › insects-2236499-supplementary.pdf]

Table S1 Collection localities, geographic and climatic parameters, host plants of *Bemisia tabaci* samples examined in this study.

No.	Code	Location	lon	Lat	AMT	AP	Host	Collect date (year, month)	Total	No. of each cryptic species							
										MED	MEAM1	AsiaI	AsiaII1	AsiaII2	AsiaII6	China1	China6
1	BJ	Changping, Beijing	116.226	40.172	11.2	595	tomato	2021.11	29	29	0	0	0	0	0	0	0
2	LNSY	Shenyang, Liaoning	123.626	41.806	8.5	725	cucumber	2021.09	18	9	9	0	0	0	0	0	0
							eggplant	2021.09	15	15	0	0	0	0	0	0	0
3	SDDZ	Dezhou, Shandong	116.428	37.164	14.1	517	tobacco	2021.08	19	19	0	0	0	0	0	0	0
							pumpkin	2021.08	18	18	0	0	0	0	0	0	0
							abutilon avicennae	2021.08	16	16	0	0	0	0	0	0	0
4	SDQD	Qingdao, Shandong	120.391	36.3072	12.3	728	eggplant	2021.11	25	25	0	0	0	0	0	0	0
5	SDBZ	Binzhou, Shandong	117.668	37.062	13.5	587	tomato	2021.08	19	19	0	0	0	0	0	0	0
6	SDLY	Laiyang, Shandong	120.712	36.964	11.4	725	cucumber	2021.07	10	4	6	0	0	0	0	0	0
							eggplant	2021.07	9	8	1	0	0	0	0	0	0
7	SDHZ	Heze, Shandong	115.577	35.106	14.2	659	eggplant	2021.10	17	17	0	0	0	0	0	0	0
							tomato	2021.10	18	18	0	0	0	0	0	0	0
8	HeNZZ	Zhengzhou, Henan	113.66	34.786	14.4	639	cucumber	2021.10	29	29	0	0	0	0	0	0	0
							cotton	2021.10	30	30	0	0	0	0	0	0	0
9	HeNXY	Xinyang, Henan	113.892	32.245	15.4	1027	eggplant	2021.08	5	0	0	0	0	2	0	2	1
10	JSJY	Jiangyan, Jiangsu	120.047	32.413	14.9	1006	eggplant	2021.08	13	13	0	0	0	0	0	0	0
11	JSYZ	Yizheng, Jiangsu	119.174	32.397	15.3	999	eggplant	2021.09	4	0	0	0	0	1	0	3	0
12	AHJX	Jixi, Anhui	118.642	30.154	15.2	1585	eggplant	2021.09	4	0	0	0	0	1	0	3	0
13	HBWX	Wuxue, Hubei	115.605	30.137	16.9	1439	cucumber	2021.09	9	2	0	0	0	2	0	5	0
							eggplant	2021.09	12	2	0	0	0	0	0	10	0

14	HBJZ	Jingzhou, Hubei	112.067	30.361	16.7	1095	cucumber	2021.08	6	0	0	0	0	0	0	6	0
							eggplant	2021.08	3	2	0	0	0	0	0	1	0
15	HBWH	Wuhan, Hubei	114.3512	30.4772	17.2	1279	tomato	2021.11	24	16	8	0	0	0	0	0	0
16	HNZZ	Yuxian, Hunan	113.398	27.145	17.9	1565	cucumber	2021.06	15	1	0	0	0	0	0	14	0
17	JXSG	Shanggao, Jiangxi	115.056	28.232	18.0	1275	tomato	2021.08	11	9	0	0	0	0	0	2	0
18	JXNC	Nanchang, Jiangxi	116.016	28.54	18.1	1132	cucumber	2021.08	8	1	0	0	0	0	0	7	0
19	ZJXS	Xiangshan,Zhejiang	121.908	29.608	16.6	1392	cucumber	2021.06	16	1	0	0	14	0	0	1	0
20	ZJCX	Cixi, Zhejiang	121.396	30.145	16.4	1316	pepper	2021.06	16	14	2	0	0	0	0	0	0
							eggplant	2021.06	16	14	1	0	0	1	0	0	0
21	ZJYY	Yuyao, Zhejiang	121.024	30.081	16.7	1288	cucumber	2021.06	11	0	0	0	3	0	0	8	0
							eggplant	2021.06	15	1	0	0	6	0	0	8	0
22	GDSZ	Shenzhen, Guangdong	114.489	22.598	22.2	2142	eggplant	2021.10	21	21	0	0	0	0	0	0	0
							tomato	2021.10	20	20	0	0	0	0	0	0	0
23	HNQH	Qionghai, Hainan	110.582	19.161	24.9	1888	cucumber	2021.03	15	0	12	0	1	0	0	2	0
24	HNSY	Sanya, Hainan	109.531	18.301	25.4	1383	cucumber	2021.09	24	0	23	1	0	0	0	0	0
							cotton	2021.09	20	16	4	0	0	0	0	0	0
25	HNLS	Lingshui, Hainan	110.041	18.442	25.6	1539	tomato	2021.03	16	2	14	0	0	0	0	0	0
26	GXNN	Nanning, Guangxi	108.33	23.04	22.2	1528	soybean	2021.09	17	0	0	9	0	0	8	0	0
27	YNLC	Lingcang, Yunnan	100.35	24.336	17.1	1067	pepper	2021.10	28	28	0	0	0	0	0	0	0
28	YNDL	Dali, Yunnan	100.2728	25.225	15.5	1010	tomato	2021.10	29	23	1	5	0	0	0	0	0
29	SCZG	Zigong, Sichuan	105.0677	29.178	18.2	1081	tomato	2021.08	15	3	0	0	0	0	0	12	0

Table S2 The primers used in this study

Organism	Target	Primer name	Primer sequences (5'→3')	Annealing temp(℃)	Reference
Bemisia tabaci	mtCOI	2195-F	TGRTTYTTTGGTCATCCVGAAGT	55	[71]
		2819-R	TTACTGCACTTTCTGCCACATTAG	55	
Portiera	16S rRNA	Por-F	GGAAACGTACGCTAATAC	58	[67]
		Por-R	TGACGACAGCCATGCAGCAC	58	
Cardinium	16S rRNA	CFB-F	GCGGTGTAAAATGAGCGTG	58	[66]
		CFB-R	ACCTMTTCTTAACTCAAGCCT	58	
Rickettsia	16S rRNA	Ric-F	GCTCAGAACGAACGCTATC	58	[28]
		Ric-R	GAAGGAAAGCATCTCTGC	58	
Hamiltonella	16S rRNA	Ham-F	TGAGTAAAGTCTGGGAATCTGG	58	[65]
		Ham-R	AGTTCAAGACCGCAACCTC	58	
Wolbachia	16S rRNA	Wol-F	TTGTAGCCTGCTATGGTATAACT	55	[68]
		Wol-R	GAATAGGTATGATTTTCATGT	55	
Arsenophonus	23S rRNA	Ars23S-1	CGTTTGATGAATTCATAGTCAAA	58	[69]

		Ars23S-2	GGTCCTCCAGTTAGTGTTACCCAAC	58	
Fritschea	23S rRNA	U23F	GATGCCTTGGCATTGATAGGCGATGAAGGA	58	[70]
		23SIGR	TGGCTCATCATGCAAAAGGCA	58	

Reference:

65. Zchori-Fein, E.; Brown, J.K. Diversity of prokaryotes associated with *Bemisia tabaci* (Gennadius) (Hemiptera: Aleyrodidae). *Ann. Entomol. Soc. Am.* **2002**, *95*, 711–718.

66. Weeks, A.R.; Velten, R.; Stouthamer, R. Incidence of a new sex-ratio-distorting endosymbiotic bacterium among arthropods. *Proc. R. Soc. Lond. B Biol. Sci.* **2003**, *270*, 1857–1865.

67. Thierry, M.; Becker, N.; Hajri, A.; Reynaud, B.; Lett, J.M.; Delatte, H. Symbiont diversity and non-random hybridization among indigenous (Ms) and invasive (B) biotypes of *Bemisia tabaci. Mol. Ecol.* **2011**, *20*, 2172-2187. https://doi.org/10.1111/j.1365-294X.2011.05087.x.

68. O'Neill, S.L.; Giordano, R.; Colbert, A.M.; Karr, T.L.; Robertson, H.M. 16S rRNA phylogenetic analysis of the bacterial endosymbionts associated with cytoplasmic incompatibility in insects. *Proc. Natl. Acad. Sci. USA* **1992**, *89*, 2699–2702. https://doi.org/10.1073/pnas.89.7.2699.

69. Thao, M.L.; Baumann, P. Evidence for multiple acquisition of *Arsenophonus* by whitefly species (Sternorrhyncha: Aleyrodidae). *Curr. Microbiol.* **2004**, *48*, 140–144. https://doi.org/10.1007/s00284-003-4157-7.

70. Everett, K.D.E.; Thao, M.; Horn, M.; Dyszynski, G.E.; Baumann, P. Novel chlamydiae in whiteflies and scale insects: Endosymbionts ‘Candidatus *Fritschea bemisiae*’ strain Falk and ‘Candidatus *Fritschea eriococci*’ strain Elm. *Int. J. Syst. Evol. Microbiol.* **2005**, *55*, 1581–1587. https://doi.org/10.1099/ijs.0.63454-0.

71. Chu, D.; Hu, X.; Gao, C.; Zhao, H.; Nichols, R. L.; Li, X. Use of mitochondrial cytochrome oxidase I polymerase chain reaction-restriction fragment length polymorphism for identifying subclades of *Bemisia tabaci* Mediterranean group. *J. Econ. Entomol.* **2012**, *105*, 242–251. https://doi.org/10.1603/ec11039.

Table S3 The infection of facultative bacterial endosymbionts in *Bemisia tabaci* MED

No.	Code	Location	Lon.	Lat.	AMT	AP	Host	Total	Infection frequency (%)						
									Car.	*Ric.*	*Ham.*	*Ric.&Him*	*Ric.&Him .&Car.*	*Car.&Ric.*	*Car.&Ham.*
1	BJ	Chanpin, Beijing	116.226	40.172	11.2	595	tomato	29	24.1	79.3	100	79.3	10.3	10.3	24.1
2	LN	Shengyang, Liaoning	123.626	41.806	8.5	725	cucumber, eggplant	24	12.5	20.8	100	20.8	0	0	12.5
3	SDDZ	Dezhou, Shandong	116.428	37.164	14.1	517	tobacco, pumpkin	53	9.4	98.1	90.6	84.9	7.5	9	7.5
4	SDQD	Qindao, Shandong	120.391	36.307	12.3	728	eggplant	25	0	92.0	100	84.0	0	0	0
5	SDBZ	Bingzhou, Shandong	117.668	37.062	13.5	587	tomato	19	0	100	73.7	73.7	0	0	0
6	SDLY	Laiyang, Shandong	120.712	36.964	11.4	725	cucumber, eggplant	12	25.0	100	75.0	58.3	0	0	0
7	SDHZ	Heze, Shandong	115.577	35.106	14.2	659	eggplant, tomato	35	11.4	97.1	88.6	74.3	8.6	11.4	8.6
8	HeNZZ	Zhenzhou, Henan	113.660	34.786	14.4	639	cucumber, cotton	59	5.1	76.3	100	76.3	0	0	5.1
9	JSJY	Jiangyan, Jiangsu	120.047	32.413	14.9	1006	eggplant	13	0	53.8	61.5	53.8	0	0	0
10	HBWH	Wuhan, Hubei	114.351	30.477	17.2	1279	tomato	16	6.3	93.8	87.5	75.0	6.3	6	6.3
11	JXSG	Shanggao, Jiangxi	115.056	28.232	18.0	1275	tomato	9	0	77.8	77.8	66.7	0	0	0
12	ZJCX	Cixi, Zhejiang	121.396	30.145	16.4	1316	pepper, eggplant	28	0	39.3	89.3	35.7	0	0	0
13	GDSZ	Shenzhen, Guangdong	114.489	22.598	22.2	2142	eggplant, tomato	41	31.7	80.5	97.6	80.5	24.4	24.4	34.1
14	HNSY	Sanya, Hainan	109.531	18.301	25.4	1383	cucumber, cotton	16	56.3	93.8	81.3	18.8	25.0	31.3	0
15	YNLC	Lingchang, Yunnan	100.350	24.336	17.1	1067	pepper	28	14.3	85.7	100	85.7	14.3	14.3	14.3
16	YNDL	Dali, Yunnan	100.273	25.225	15.5	1010	tomato	23	8.7	69.6	100	69.6	4.3	4.3	8.7

Table S4 The infection of facultative bacterial endosymbionts in *Bemisia tabaci* MEAM1

No.	Code	Location	Lon.	Lat.	AMT	AP	Host	Total	Infection frequency (%)			
									Car.	*Ric.*	*Ham.*	*Ric.&Him*
1	ZJCX	Cixi, Zhejiang	121.396	30.145	16.4	1316	pepper, eggplant	3	0	66.7	66.7	66.7
2	SDLY	Laiyang, Shandong	120.712	36.964	11.4	725	cucumber, eggplant	7	0	71.4	0	0
3	HNLS	LinShui, Hainan	110.041	18.442	25.6	1539	tomato	14	0	71.4	100	71.4
4	HNQH	Qionghai, Hainan	110.582	19.161	24.9	1888	cucumber	12	0	16.7	66.7	16.7
5	HNSY	Sanya, Hainan	109.531	18.301	25.4	1383	cucumber, cotton	27	0	7.4	74.1	7.4
6	LNSY	Shengyang, Liaoning	123.626	41.806	8.5	725	cucumber, eggplant	9	0	55.6	66.7	33.3
7	HBWH	Wuhan, Hubei	114.351	30.477	17.2	1279	tomato	8	0	75.0	87.5	75.0

Table S5 The infection of facultative bacterial endosymbionts in native species of *Bemisia tabaci*

No.	Code	Location	Lon.	Lat.	AMT	AP	Host	Native species	Total	Infection frequency (%)			
										Car.	*Ric.*	*Ham.*	*Ric.&Ham.*
1	HNXY	Xinyang, Henan	113.892	32.245	15.4	1027	tobacco	Asia II 2(2), China 1(2)，China 6(1)	5	40.0	20.0	40.0	0
2	AHJX	Jixi, Anhui	118.642	30.154	15.2	1585	eggplant	Asia II 2(1), China 1(3)	4	0	25.0	0	0
3	HBJZ	Jingzhou, Hubei	112.067	30.361	16.7	1095	cucumber, eggplant	China 1(7)	7	0	14.3	0	0
4	HNZZ	Zuzhou, Hunan	113.398	27.145	17.9	1565	cucumber	China 1(14)	14	0	21.4	0	0
5	JXSG	Shanggao, Jiangxi	115.056	28.232	18.0	1275	tomato	China 1(2)	2	0	50.0	0	0
6	ZJYY	Yuyao, Zhejiang	121.024	30.081	16.7	1288	cucumber, eggplant	Asia II 1(9)	9	0	8.0	0	0
7	HNQH	Qionghai, Hainan	110.582	19.161	24.9	1888	cucumber	Asia II 2(1), China 1(2)	3	0	100	33.3	33.3
8	GXNN	Nanning, Guangxi	108.330	23.040	22.2	1528	soybean	Asia I (9), Asia II 6(8)	17	0	5.9	11.8	0
9	YNDL	Dali, Yunnan	100.273	25.225	15.5	1010	tomato	Asia I (5)	5	0	4.0	0	0

Table S6 Infection frequency of facultative bacterial endosymbionts in *Bemisia tabaci* cryptic species

Bemisia tabaci cryptic species	Locations and numbers	Infection frequency (%)						
		Por.	*Car.*	*Ric.*	*Ham.*	*Wol.*	*Ars.*	*Fri.*
Asia I	HNSY (1)	100	0	0	0	0	0	0
	GXNN (9)	100	0	11.11	0	11.11	0	0
	YNDL (5)	100	0	60	0	0	0	0
Asia II 1	ZJXS (14)	100	0	0	0	0	7.1	0
	ZJYY (9)	100	0	11.11	0	0	0	0
Asia II 2	HeNXY (2)	100	100	0	0	0	0	0
	JSYZ (1)	100	0	0	0	0	0	0
	AHJX (1)	100	0	0	0	0	0	0
	HBWX (2)	100	0	0	0	0	0	0
	ZJCX (1)	100	0	0	0	0	0	0
	HNQH (1)	100	0	100	0	100	0	0
Asia II 6	GXNN (8)	100	0	0	0	25	0	0
China 6	HeNXY (1)	100	0	0	0	0	0	0
China 1	HeNXY (2)	100	0	0	0	0	0	0
	JSYZ (3)	100	0	0	0	0	0	0
	AHJX (3)	100	0	0	0	66.67	0	0
	HBWX(15)	100	0	0	0	0	0	0
	HBJZ(7)	100	0	0	0	14.28	0	0
	HNZZ(14)	100	0	21.42	0	0.07	0	0
	JXSG(2)	100	0	50	0	50	0	0
	JXNC(7)	100	0	0	0	57.14	0	0

	ZJXS(1)	100	0	0	0	0	0	0
	ZJCX(16)	100	0	0	0	0	0	0
	HNQH(2)	100	0	100	50	0	0	0
	SCZG(12)	100	0	0	0	0	0	0
MED	BJCP(29)	100	24.10	79.30	100	0	0	0
	LNSY(24)	100	12.50	20.80	100	0	0	0
	SDDZ(53)	100	9.40	98.10	90.60	0	0	0
	SDQD(25)	100	0	92.00	100	0	0	0
	SDBZ(19)	100	0	100	73.70	0	0	0
	SDLY(12)	100	25	100	75.00	0	0	0
	SDHZ(35)	100	11.40	97.10	88.60	0	0	0
	HeNZZ(59)	100	5.10	76.30	100	0	0	0
	JSJY(13)	100	0	53.80	61.50	0	0	0
	HBWH(16)	100	6.30	93.80	87.50	0	0	0
	JXSG(9)	100	0	77.80	77.80	0	0	0
	ZJCI(28)	100	0	39.30	89.30	0	0	0
	GDSZ(41)	100	31.70	80.50	97.60	0	0	0
	HNSY(16)	100	56.30	93.80	81.30	0	0	0
	YNLC(28)	100	14.30	85.70	100	0	0	00
	YNDL(23)	100	8.70	69.60	100	0	0	0
MEAM1	ZJCX	100	0	66.70	66.70	0	0	0
	SDLY	100	0	71.40	0	0	0	0
	HNLS	100	0	71.40	100	0	0	0
	HNQH	100	0	16.70	66.70	0	0	0
	HNSY	100	0	7.40	74.10	0	0	0
	LNSY	100	0	55.60	66.70	0	0	0

	HBWH	100	0	75.00	87.50	0	0	0
	YNDL	100	0	0	100	0	0	0
